# Cortical Plasticity and Olfactory Function in Early Blindness

**DOI:** 10.3389/fnsys.2016.00075

**Published:** 2016-08-30

**Authors:** Rodrigo Araneda, Laurent A. Renier, Philippe Rombaux, Isabel Cuevas, Anne G. De Volder

**Affiliations:** ^1^Institute of Neuroscience (IoNS), Université catholique de LouvainBrussels, Belgium; ^2^Department of Otorhinolaryngology, Cliniques Universitaires Saint-LucBrussels, Belgium; ^3^Laboratorio de Neurociencias, Escuela de Kinesiología, Facultad de Ciencias, Pontificia Universidad Católica de ValparaísoValparaíso, Chile

**Keywords:** olfactory perception, congenital blindness, functional neuroimaging, cross-modal plasticity, visual deprivation, olfaction

## Abstract

Over the last decade, functional brain imaging has provided insight to the maturation processes and has helped elucidate the pathophysiological mechanisms involved in brain plasticity in the absence of vision. In case of congenital blindness, drastic changes occur within the deafferented “visual” cortex that starts receiving and processing non visual inputs, including olfactory stimuli. This functional reorganization of the occipital cortex gives rise to compensatory perceptual and cognitive mechanisms that help blind persons achieve perceptual tasks, leading to superior olfactory abilities in these subjects. This view receives support from psychophysical testing, volumetric measurements and functional brain imaging studies in humans, which are presented here.

## Introduction

The plasticity of the human brain, that is, its ability to adapt to environmental constraints by creating changes in its connectivity (synaptic plasticity) and/or in the neurons themselves (e.g., neurogenesis), is one of its most outstanding properties (Rakic, 2002). This plasticity is most striking in the sensory systems, which provide all inputs to the brain (Pascual-Leone et al., 2005). The plasticity of sensory brain areas in the cerebral cortex is the basis for the adaptability of the brain to the environment. This neural plasticity is particularly high during development, which is necessitated by the growth of the organism and the need of the brain to get programmed. The “neural Darwinism” theory predicts that in the lack of one sensory modality, as in congenital blindness, the target structures are taken over by the afferent inputs from other senses that will promote and control their functional maturation (Edelman, 1993). Numerous studies have shown the effects of early visual deprivation on the development of the remaining senses (e.g., Van Boven et al., 2000; Goldreich and Kanics, 2003, 2006; Gougoux et al., 2004; Voss et al., 2004; Wan et al., 2010) and higher cognitive functions (e.g., Amedi et al., 2003; Röder and Rösler, 2003; Pasqualotto et al., 2013). The emergence of behavioral adjustments in early blindness has been usually associated with the functional reorganization of the deafferented visual cortical areas (i.e., occipital brain areas), which are recruited to process non-visual information (Amedi et al., 2003; Gougoux et al., 2005; Ricciardi et al., 2007; Kupers et al., 2010; Renier et al., 2010; Collignon et al., 2011; Kitada et al., 2014; Bedny et al., 2015; Dormal et al., 2016). Most of these previous studies were focused on tactile and auditory functions as well as on higher cognitive processing such as auditory memory and language operations. Although blind individuals rely extensively on touch and audition to get environmental information (Hatwell, 2003), they pay attention to *all* non-visual cues, including the odors (Ferdenzi et al., 2010). Olfactory processing might contribute to the multisensory tuning that takes place during development and perceptual learning in subjects with early-onset blindness (Proulx et al., 2014). The present article deals with the behavioral, anatomical and physiological plasticity in the treatment of olfactory information in humans that grow up blind. Here, we summarize the observations providing evidence for compensatory mechanisms at a sensory level (i.e., odor detection) and basic level of perception (i.e., odor discrimination), as well as in higher order perceptual processes and cognitive adjustments in olfaction (i.e., odor categorization integrating semantic aspects of odor identification). Psychophysical testing of olfactory performance is described in relation with anatomical changes in olfactory bulb (OB) volumetric measurements assessed by MRI. Besides behavioral changes and practice-related adjustments due to early blindness, we discuss the functional brain reorganization as another factor influencing olfactory perceptual skills in early blind (EB) individuals, as well as the possible underlying mechanisms for brain plasticity and olfactory function.

## Behavioral Adjustments in Olfactory Processing in Case of Early Visual Deprivation

**Figure 1:**
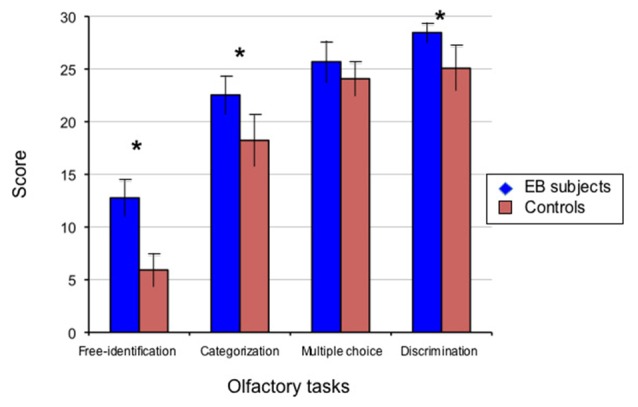
**Performance of early blind (EB) subjects and controls in psychophysical olfactory testing.** The perceptual aspects of olfaction were investigated in 13 subjects with early-onset blindness (EB) and 13 sighted controls (SCs) studied blindfolded, using a set of 30 commercially available bottles that contained microencapsulated granules of odorants selected by a perfumer (http://www.sentosphere.fr). Odorants (flower, fruit, plant or domestic elements) were presented orthonasally in one single session. A discrimination task and an identification task with three levels of cuing, i.e., free identification (no cue), categorization (semantic cue), multiple choice (semantic and phonological cues) were used to assess the olfactory abilities. In each task, the quotation was made on a 0/1 basis (for each trial in the related task, 0: wrong; 1: correct), with the total number of correct responses providing the score (maximum: 30/30). Histograms display the mean values and standard deviations of these scores for EB subjects and matched SCs as indicated. The group difference was significant (*p < 0.05) in all conditions except multiple choice identification. EB subjects significantly outperformed the SC participants in odor discrimination (*p* < 0.0002), free-identification (*p* < 0.0001) and categorization (*p* < 0.0004). The multiple choice identification scores showed a trend to a slightly better performance in the EB subjects compared to the blindfolded controls, though not significant (*p* = 0.063). Adapted from Cuevas et al. (2009).

Studying early-onset blindness represents a unique way to investigate how the absence of visual input during critical developmental periods for establishing connections does affect the functional organization of the human brain in order to achieve the best behavioral outcome. Typically, people are considered as early-onset blind when affected by total blindness (without residual light perception) as the result of bilateral ocular or optic nerve lesions established at birth or within the first 2 or 3 years of life, before the completion of visual development. Numerous behavioral studies in the auditory and tactile domains have provided evidence of the better performance of participants with early-onset blindness compared with sighted controls (SCs; Renier et al., 2014). However, the evidence concerning the sense of smell is significantly lower. One may postulate that persons with early-onset blindness rely more extensively on their olfaction than those who are sighted. For example, when vision is lacking, the olfactory sense has an enhanced ecological value for the detection of odors that yield information about the environment and for the evaluation of the quality of food (Ferdenzi et al., 2004, 2010). It might also serve as landmarks in navigation and thus contribute to spatial cognition, which is impaired in the absence of visual experience and must be balanced by perceptual learning via practice (Pasqualotto and Proulx, 2012). Although there is evidence that individuals with congenital or early-onset blindness would not perform differently from sighted in the main basal chemosensory tasks and in odor detection (Smith et al., 1993; Diekmann et al., 1994), previous works provided demonstration that EB participants do better than their age-matched controls when olfactory identification tasks are more complex, e.g., in free identification of odors (Murphy and Cain, 1986; Rosenbluth et al., 2000). This is particularly true when semantic components are involved in the task, e.g., tasks including odor name retrieval from semantic memory for odors (Wakefield et al., 2004). In a study conducted by Cuevas et al. (2009), where 13 EB participants were compared to 13 SCs matched for age, sex and handedness, EB participants did significantly better than the SCs in free identification of odors. They also outperformed the SCs, albeit to a slightly less extent, in odor categorization and discrimination (Figure 1, see also Table 1). In this study, EB participants mainly outperformed the sighted when olfactory tasks involved higher order components of odor recognition such as semantic memory aspects. There was no group difference when participants were requested to identify each odor by selecting its name from a list of propositions (multiple forced-choice identification). This could be interpreted as EB individuals being less dependent on the use of provided semantic and phonological information to name odors when compared to sighted subjects (who had a similar performance as EB subjects in the multiple choice condition whereas they performed very poorly when no information was provided). This is in accordance with previous observations of enhanced abilities by the blind when complex olfactory identification tasks were assessed, e.g., in free identification (Murphy and Cain, 1986; Rosenbluth et al., 2000). Nevertheless, using a set of standardized psychophysical tests (the Sniffin’ Sticks, Hummel et al., 2007), Cuevas et al. (2010b) assessed three components of olfactory acuity in EB subjects and controls matched for age, sex, and handedness: odor detection threshold (T), odor discrimination (D) and odor identification (I) from a list of four descriptors (multiple forced-choice). When the two groups were compared for the composite (T + D + I) score there was a significant difference that was mainly due to better scores for odor detection, and to a lesser extent, better odor discrimination by the blind (Table 2). This indicated that EB people developed compensatory changes in the olfactory perception domain that also involved basic sensory processes, such as a lower threshold for odor detection and slightly better odor discrimination. Interestingly, in the group of EB subjects the best composite (T + D + I) scores were observed in the older participants, whereas we observed the reverse pattern in the SC group.

**Table 1:** **Results from behavioral experiment in blind and sighted subjects (Cuevas et al., 2009)**.

Subjects		Age (years)	Odor discrimination (score/30)	Odor free-identification (score/30)	Odor categorization (score/30)	Multiple identification choice (score/30)
EB1	(*)	23	30	16	22	27
EB2	(*)	28	29	13	25	25
EB3	(*)	31	28	11	20	24
EB4	(*)	42	28	12	21	29
EB5	(*)	57	30	15	21	26
EB6	(*)	31	28	14	24	26
EB7	(*)	43	29	10	20	22
EB8	(*)	40	29	14	25	28
EB9	(*)	52	28	14	23	26
EB10	(*)	48	27	11	23	27
EB11		23	28	11	21	27
EB12		21	27	13	25	23
EB13		23	29	12	23	24
Mean (EB)		35.5	28.5	12.8	22.5	25.7
SD (EB)		12.2	0.97	1.79	1.85	2.02
SC1	(*)	22	26	8	20	24
SC2		28	27	8	17	24
SC3	(*)	31	28	7	21	26
SC4	(*)	42	25	8	13	23
SC5		55	25	5	18	24
SC6	(*)	29	27	5	21	26
SC7	(*)	42	21	5	18	23
SC8	(*)	41	27	7	18	21
SC9	(*)	51	24	7	22	22
SC10	(*)	48	25	4	19	27
SC11		24	25	4	18	24
SC12		22	21	4	17	24
SC13		22	25	5	15	25
Mean (SC)		35.2	25.1	5.9	18.2	24.1
SD (SC)		11.9	2.14	1.61	2.49	1.66

Note: EB, early blind; SC, sighted control; (*): subject who also participated in the neuroimaging study with fMRI (see Figure 4); all subjects were right-handed males; sighted subjects were studied blindfolded. Behavioral performance (number of correct answers) was assessed in a variety of higher-level odor processing tasks using a set of 30 bottles that contained microencapsulated granules of odorants presented orthonasally. A discrimination task and an identification task with three levels of cuing, i.e., free identification (no cue), categorization (semantic cue), multiple choice (semantic and phonological cues) were used to assess the olfactory abilities. The group difference was significant in all conditions except multiple choice identification (see Figure 1). Data from Cuevas et al. (2009).

**Table 2:** **Results from behavioral experiment in blind and sighted subjects (Cuevas et al., 2010b)**.

Subjects	Age (years)	Odor threshold (score/16)	Odor discrimination (score/16)	Multiple choice identification (score/16)	TDI composite score (score/48)	Retronasal testing (score/20)
EB1	21	7.3	15	10	32.3	19
EB3	29	6.3	10	16	32.3	16
EB4	40	7.0	14	14	35.0	17
EB5	55	15.8	15	13	43.8	18
EB8	39	11.0	16	11	38.0	17
EB9	51	8.0	14	15	37.0	17
EB10	45	15.8	13	14	42.8	18
EB11	20	6.0	15	12	33.0	18
Mean (EB)	37.5	9.6	14.0	13.10	36.8	17.5
SD (EB)	13.1	4.08	1.85	2.03	4.54	0.93
SC1	20	3.5	14	16	33.5	20
SC5	53	5.0	9	14	28.0	19
SC7	40	6.3	9	13	28.3	16
SC8	39	6.0	14	11	31.0	19
SC9	50	4.8	11	13	28.8	17
SC14	21	4.5	14	14	32.5	17
SC15	30	6.3	13	14	33.3	16
SC16	39	4.0	10	14	28.0	18
Mean (SC)	36.5	5.0	11.8	13.6	30.4	17.8
SD (SC)	12.1	1.05	2.25	1.41	2.43	1.49

*Note: EB, early blind; SC, sighted control (same numbering as in Table 1); all subjects were right-handed males; sighted subjects were studied blindfolded. Scores were for odor detection threshold (T), odor discrimination (D) and multiple forced-choice identification (I) in the Sniffin’ Stickx test (Hummel et al., 2007). The TDI composite score referred to the sum of individual results obtained for these three subtests (threshold + discrimination + multiple forced choice identification). The retronasal testing (based on the protocol of Heilmann et al., 2002) referred to a multiple forced-choice identification of odorized powders presented via the mouth. For odor detection threshold (ranging from 1 to 16), the closer to 16 corresponded to the higher sensibility to odors (corresponding to a low threshold) and conversely. For all other measures, the score was the sum of correct responses (with a quotation made on a “0: wrong; 1: correct” basis). The EB participants outperformed the SC subjects in the odor detection task (*p* = 0.007, lower detection threshold in EB) and to a lesser extent, in odor discrimination (*p* = 0.03) but not in odor multiple forced-choice identification, either orthonasally (*p* = 0.85), nor retronasally (*p* = 0.75). The composite TDI score was significantly different between groups (*p* = 0.0032), mainly due to better scores in EB for odor detection. Data from Cuevas et al. (2010b)*.

Standard psychophysical testing of olfaction, such as the clinical Sniffin’ Sticks test, is generally intended to assess orthonasal olfactory function, i.e., presenting odors on felt-tip pens in front of the nostrils for birhinal stimulation, which is particularly useful to detect sinonasal disease (Rombaux et al., 2006c). There is increasing interest to also include retronasal testing in the clinical evaluation of olfactory function (Rombaux et al., 2006c). Retronasal olfaction is assessed using odorized powders or granules applied in the oral cavity, i.e., applying each sample to the midline of the tongue to allow odor processing, after which the participant is asked to rinse his/her mouth abundantly with water (after each single-substance test). It is noteworthy that the two groups in the study of Cuevas et al. (2010b) did not differ from each other when odors were presented via the retronasal way, using odorized granules applied to the tongue (Table 2). Using a battery of 38 odorant chemicals, Gagnon et al. (2015a) recently demonstrated that congenitally blind participants tended to be better and were significantly faster at identifying odors presented orthonasally whereas this was not observed when odorants were presented retronasally. Their data revealed that early-onset blind subjects “were more familiar with the orthonasal odors and used the retronasal odorants less often for cooking than their sighted counterparts”, a result that is concordant with a reduced food variety exposure in this group, which might also explain their reduced taste perception when compared to SCs (Gagnon et al., 2014, 2015b). In another study by the same group (Beaulieu-Lefebvre et al., 2011) a significantly lower odor detection threshold was observed in EB participants compared to SCs. However, no group differences were observed either for odor discrimination or multiple forced-choice identification when comparing congenitally blind and SC subjects using the Sniffin’ Sticks test. In addition, blind participants scored higher when evaluated for their consciousness of olfactory sensations, as assessed by the Odor Awareness Scale (OAS). This brought further support to the hypothesis according to which congenitally blind subjects would use more their sense of smell than sighted subjects in their daily life, i.e., in order to assess their environment and to recognize places and other people (Iversen et al., 2015). A recent study conducted by Çomoğlu et al. (2015) compared participants with congenital blindness, acquired blindness and sighted subjects using the Sniffin’ Sticks test. In this study, the blind group (with congenital and acquired blindness pooled together) was significantly better in odor detection and discrimination, but not in multiple-forced choice identification. In addition, in this study there were no differences between participants with congenital and acquired blindness in any assessment. Together, these behavioral studies, despite methodological differences, indicate that compared to the sighted, EB persons roughly present a better olfactory performance, especially when semantic aspects are involved in the task (i.e., in free identification of odors) but also at a more basic level (i.e., as reflected by a lower threshold for odor detection). This is probably due to practice-related abilities, because in the blind the sense of smell contributes significantly in the assessment and perception of the environment. The neural plasticity that underlay these results may involve peripheral and central mechanisms.

## Anatomical Reorganization of Olfactory System in Response to Early Visual Deprivation

**Figure 2:**
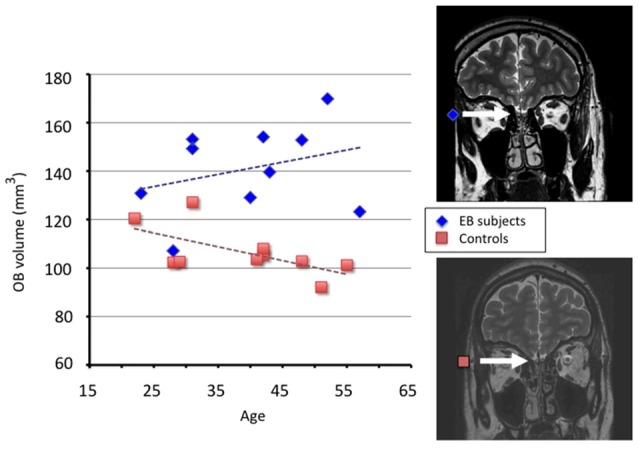
**Results from measurements of olfactory bulb (OB) volume in EB subjects and controls.** Using a 3-Tesla MRI and a T2-weighted fast spin-echo sequence in 10 male subjects with early blindness and 10 matched controls, individual OB volume was calculated by plannimetric manual contouring on 23 coronal slices (1.5 mm thickness) perpendicular to the cribriform plane and covering the middle segment of the basifrontal area. Measurements were taken twice by two observers and the mean of these measurements was included as the definite volume, according to a validated protocol for OB analysis (Rombaux et al., 2009 and references therein). The OB volume (right + left) in mm^3^ is plotted as a function of age in EB subjects (blue diamonds, *r* = 0.30) and controls (orange squares, *r* = −0.62). Coronal T2 sequence MRI scans of a representative EB subject (top) and a control (bottom) are displayed at the level of OB (indicated by the white arrow). OB: occipital bulb; EB: early blind. Adapted from Rombaux et al. (2010).

To compensate for their lack of vision, subjects with early-onset blindness develop enhanced abilities in the use of their remaining senses, hypothetically because of a cross-modal reorganization of deafferented visual brain circuitry to process non visual information such as sounds or tactile stimuli (Röder et al., 1999; Gougoux et al., 2004). In addition, an increasing number of studies report intramodal brain changes, for example in the auditory regions (Elbert et al., 2002; Stevens and Weaver, 2009). In the olfactory system, the OB is the first relay in which olfactory information is processed, playing a key role in the human olfactory function (Mori et al., 1999). The volume of the OB changes as a function of olfactory performances and training, as well as when recovering from chronic olfactory dysfunction (Rombaux et al., 2009). A study conducted by Rombaux et al. (2010) evaluated the OB volume measured on MRI scans and the olfactory function in 10 EB participants compared with 10 SCs who were matched for age, sex and handedness. Although based on a relatively small but well selected study population, this study highlighted the increase in the OB volume in EB participants (Figure 2), in whom the odor discrimination and free identification tasks yielded higher scores compared with the controls. The difference in the mean (right + left) OB volume between EB and control subjects was 34.3 mm^3^ (95% confidence interval (CI): 20.3–48.4 mm^3^, *p* = 0.0001). This result is in accordance with the earlier morphometric studies reporting that although regions along the visual pathways are atrophied in people with early-onset blindness, some brain areas outside the occipital cortex may be hypertrophied, indicating widespread compensatory adaptations (Pan et al., 2007; Fortin et al., 2008; Hasson et al., 2016).

Since blind subjects get the environmental data by using the senses of touch and hearing and olfaction, the blind subject’s enhanced olfactory abilities are, at least partly practice-related. Enhancement in olfactory function and increase of OB volume in EB subjects may occur via the development of new synapses. Brain-derived neurotrophic factor (BDNF) is a neurotrophic protein that facilitates the growth and differentiation of new synapses and could be a mediator of OB adaptations to the visual deprivation. At least, it has been shown that the over-expression of BDNF raises the OB granule cell dendritic spine density in mice (McDole et al., 2015). The activity-dependent nature of BDNF expression makes it ideally suited to mediate the trophic effects of practice-related activity on neuronal maturation and synaptic plasticity. However, before claiming this, studies are needed to assess the neurotrophic role of BDNF, which may be down-regulated by chronic stress and up-regulated by learning processes and physical activity, so that the serum level of BDNF may be affected by many confounders. For instance, although a better odor detection threshold and superior odor discrimination have been described in blind people, including in those with acquired blindness (Çomoğlu et al., 2015), these olfactory abilities are usually found to be worse in patients with age-related macular degeneration (Kar et al., 2015 and references therein), one of the major causes of acquired blindness, whereas the serum level of BDNF in these patients was found to be higher than that in healthy individuals (Afarid et al., 2015).

## Functional Neuroimaging of Odor Processing in Blind Humans

Apart from behavioral studies, modern neuroimaging has contributed to a better understanding of sensory and cognitive processes in case of early visual deprivation. One of the first imaging studies in blind people was performed over 20 years ago by Veraart et al. (1990), who found that the early visually deprived cortex displayed metabolic activity that was actually higher, on average, than in blindfolded SCs (Wanet-Defalque et al., 1988; see also Uhl et al., 1991). In EB adults, affected by pregeniculate (ocular or optic nerve) lesions from birth or in the first years of life, rates of glucose metabolism measured in primary and association visual cortex by means of positron emission tomography (PET) reached a level similar to that of control subjects who were studied with their eyes open (Veraart et al., 1990). These results were later substantiated by a number of studies from several laboratories that showed specific activation of occipital cortex in the blind by non visual stimuli, including Braille, tactile shapes, spoken words and sounds (Sadato et al., 1996; Büchel et al., 1998; De Volder et al., 1999; Weeks et al., 2000; Arno et al., 2001; Burton et al., 2002; Röder et al., 2002). While cross-modal plasticity in auditory and tactile modalities has been thoroughly investigated in the blind population, the question remained whether a crossmodal reorganization of deafferented visual brain areas contributed to odor processing in the EB as it was observed for tactile and auditory processing. As an attempt to gain insight into this question, studies using brain investigation techniques in condition of physiological activation were aimed to verify whether the occipital cortex of EB subjects was or was not recruited during olfactory information processing and under which condition: in low level information processing or when perception included cognitive components.

A study conducted by Cuevas et al. (2011) using chemosensory event-related potentials (ERPs) investigated the possible effect of early-onset blindness on the electrophysiological correlates of passive odor perception in EB and SC subjects matched for age, sex and handedness. This study examined the latencies, amplitudes and topographical distributions of olfactory and trigeminal event-related potentials showing no major difference between groups in accordance with a previous electrophysiological study (Schwenn et al., 2002). The absence of group difference could be due to the fact that the occipital cortex of EB subjects is functionally recruited mainly during a higher order perceptual processing and not during a basic level of odor perception (“passive stimulation”). Previous studies in early blindness supported this hypothesis for audition and touch (Weeks et al., 2000; Gizewski et al., 2003).

**Figure 3:**
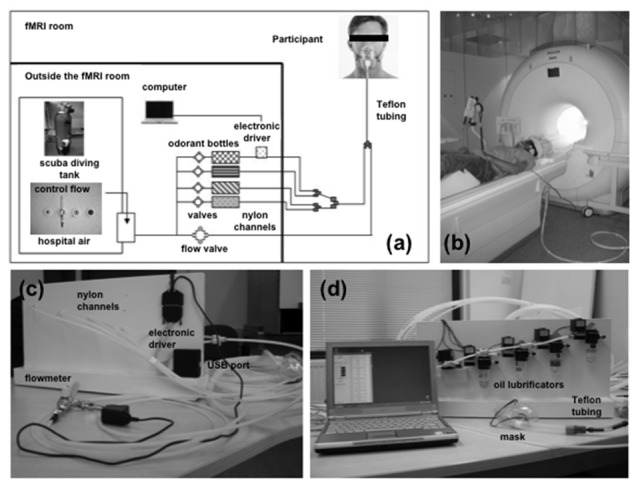
**Images of the experimental setup for fMRI study of olfactory processing. (A)** Schematic representation of the computer-controlled, MRI-compatible odor delivery system. Outside and partly inside the fMRI room, the five nylon channels transmit odorless pulsed air and odorants in separate ways, until they reach the last 30-cm segment nearest to the participant; these channels converge into a single Teflon tube connected to a mask. Outside of the fMRI room, compressed air—either from a scuba-diving tank or a hospital air-care delivery (constant flow)—provides a clean air supply for the stimulator. Bottles containing the odorants (lemon, banana, lavender, rose) are kept in the odor delivery system. An electronic driver is located in the back of the stimulator device (represented schematically in the figure). The computer that controls the stimulator device is located outside the fMRI room. **(B)** Image of a volunteer participating in a fMRI experiment using the odor delivery system. Auditory signals that allow synchronization of breathing with odor stimulations are delivered via headphones. **(C,D)** Overall view of the computer-controlled stimulator device, showing nylon channels, fittings, and Teflon tube that deliver the switched air streams to the participant via a removable medical mask; panel **(C)** shows the view from the back, showing the flowmeter, the start of the five nylon channels, the main power, and the electronic driver, which is equipped with a USB port; panel **(D)** gives a detailed front view of the device, showing the solenoid valves and oil lubrificators containing the odors in solution. The main part of the device and the computer remain outside the fMRI room, whereas the five nylon channels are passed to the fMRI room through a conventional security hole. Adapted from Cuevas et al. (2010a).

**Figure 4:**
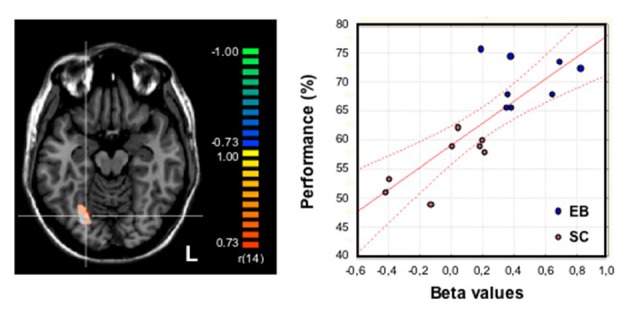
**Relationship between olfactory performance and brain activity during odor processing (odor discrimination and categorization).** Activation maps were obtained from an analysis of covariance on olfactory conditions plotted together in 16 (8 EB) subjects using their averaged performance in odor free-identification, categorization and discrimination as the covariate (the ability to discriminate, categorize and identify 30 odor samples was assessed before the fMRI study and further averaged to provide a global odor recognition performance expressed in percentage). Brain regions with a positive covariation (*p* = 0.001 uncorrected, with a cluster size threshold correction of *p* = 0.05 based on Monte Carlo simulation) were superimposed on the transversal view of a normalized MRI brain of a representative subject. An activation focus was found in the right fusiform gyrus [FG, in orange-yellow: 380 mm^3^ (center of gravity: *x*: 24, *y*: −64, *z*: −13)] that largely overlapped a brain area that had been identified in the group comparison (EB − SC) for olfactory processing and displayed here in white color as a reference. The crosshairs intersect on a voxel in the right FG (*x*: 24, *y*: −67, *z*: −13). The graph at the right part of the figure shows the strong correlation between brain activity (beta weights) in the right FG during odor processing (white region) and the individual performance (averaged score, %) in the whole group of subjects (*n* = 16): *r* = 0.80; *p* = 0.001. The red lines indicate the confidence interval (CI, 95%). EB, early blind; SC, sighted control (studied blindfolded); L, left hemisphere. Adapted from Renier et al. (2013).

Brain activation studies carried out with fMRI proved that several regions of the occipital cortex were recruited during active conditions of olfactory processing in humans affected by early blindness. An fMRI study conducted by Kupers et al. (2011) showed that congenitally blind subjects had augmented central responses during odor processing. Compared to SC subjects, congenitally blind subjects activated more strongly a subset of the primary (right amygdala) and higher order olfactory areas, such as the lateral orbitofrontal cortex in the right hemisphere, the mediodorsal thalamus and the hippocampus bilaterally. Given the better scores for odor detection and awareness in congenitally blind subjects (Beaulieu-Lefebvre et al., 2011), the activity observed in olfactory areas was attributed to an increment in odor processing associated with attention or emotional process. Noteworthy, blind subjects also showed a stronger recruitment of their occipital cortex, mainly in V2, during the odor detection task, suggesting a privileged access of olfactory stimuli to this cortex when visually deafferented from birth (Kupers et al., 2011; Kupers and Ptito, 2014). Interestingly, while EB subjects, who had increased sensitivity to orthonasal odorants, recruited their visually deprived occipital cortex to process orthonasal olfactory stimuli in a simple odor detection task, they did not recruit their occipital cortex to process taste stimuli (Gagnon et al., 2014, 2015b). This was consistent with behavioral observations, since EB performed less well than SCs in taste and retronasal olfaction (Gagnon et al., 2015a), i.e., when processing chemicals inside the mouth. Our own study (Renier et al., 2013) aimed to investigate the cerebral network supporting performance in higher-level odor processing and to compare, in the same blind subjects, the brain activity elicited by stimuli processed in different sensory modalities. Using fMRI and an MRI-compatible odor delivery system (Figure 3) in ten participants with early-onset blindness, we observed a strong activation of the occipital cortex during two olfactory processing tasks (discrimination or categorization of fruit and flower odors), as well as during control auditory-verbal conditions (discrimination or categorization of fruit and flower names). We also observed a functional dissociation between olfactory and auditory-verbal processing in the occipital cortex of EB subjects; the right fusiform gyrus (FG) was most activated during the olfactory conditions and part of the left middle occipital gyrus, located in the posterior part of the ventral lateral occipital complex showed a preference for auditory-verbal processing (Renier et al., 2013). In addition, there was a strong correlation between the level of right FG activation during the olfactory conditions and the individual performance averaged from a variety of odor recognition tests (Figure 4). Only little occipital activation was observed in SCs, but the same “right olfactory/left auditory-verbal” hemispheric lateralization was found overall in their brain. This dissociation was observed independently to the task performed (e.g., stimulus discrimination and stimulus categorization), which indicates that it was mainly stimulus-driven.

Taken together, all these evidences indicate that, as in the auditory and tactile modalities, cross-modal plasticity was observed using olfactory stimuli in the blind population. However, the recruitment of visually deprived occipital areas was mainly observed when perception included cognitive components and not when low level information processing was evaluated. These findings constituted new evidence in favor of a functional specialization in the occipital cortex of EB people, shedding light on how non-visual modalities including olfaction are distributed in their reorganized occipital cortex.

## Behavioral Adjustments and Functional Brain Reorganization in Early Blindness: Possible Underlying Mechanisms in Olfactory Function

The sense of smell is highly plastic and depends on learning and experience (Wilson and Stevenson, 2003). EB individuals are in a “special” sensory context where they make an extensive use of their remaining senses, including olfaction (Ferdenzi et al., 2010). The previous work provided evidence that EB subjects develop superior olfactory abilities both at the sensory (i.e., odor detection) and basic perceptual level (i.e., odor discrimination) and as well at a higher-order cognitive level (i.e., odor free-identification and categorization). In addition to practice-related adjustments, there is a cross-modal recruitment of the occipital cortex in EB subjects, as shown using fMRI, that supports their superior perceptual-cognitive olfactory skills. However, this intermodal reorganization of visually deprived brain areas does not appear to support behavioral changes at the sensory level, since in the study using the chemosensory brain potentials technique “passive” olfactory stimulation without task did not elicit higher brain responses in the occipital cortex of EB subjects compared to controls (Cuevas et al., 2011). Actually, normal brain responses were observed in their cortex as well as similar chemosensory ERPs components as those observed in previous studies (Murphy et al., 2000; Rombaux et al., 2006a), leading to hypothesize that olfactory stimulus detection, although not overtly required, was similarly present in both groups. This study did not provide a definite answer to the question “how could we explain the superior olfactory abilities at the sensory level in EB subjects”?

It is well known that the functional brain reorganization after early visual deprivation does not only include intermodal plastic changes, but also intramodal plasticity (Merabet and Pascual-Leone, 2010). In the olfactory system, the olfactory epithelium and the OBs are the structures related to odor detection (i.e., olfactory sensitivity), transduction and encoding of olfactory information (Hummel and Welge-Lüssen, 2006). From a structural and functional point of view, some authors affirm that “the OB represents the first level of olfactory information processing in the brain” (Mori et al., 1999; Laskaris et al., 2008). It has been shown that the OB has a high degree of plasticity in response to olfactory experience (Mandairon and Linster, 2009) and treatment of olfactory disorders (Gudziol et al., 2009). In addition, the OB undergoes volume changes as a function of olfactory performance (Rombaux et al., 2006b; Haehner et al., 2008). Taking these observations into account, it is logical to think that the OB of EB individuals could undergo structural changes and even may develop hypertrophy in response to their enhanced use in olfactory information processing, as observed using the MRI volumetric technique (Rombaux et al., 2010). This particularity could be interpreted as reflecting intramodal adjustments in EB subjects that could explain the better olfactory sensitivity observed in this population at the sensory level. In contrast to the study of Kupers et al. (2011), our own fMRI study did not provide evidence of intramodal plasticity, neither in primary or secondary olfactory cortex of EB subjects (Renier et al., 2013). Perhaps this was due to the fact that we used higher-level odor processing conditions with cognitive components, which would rather show up crossmodal plasticity changes in this population.

Another central question concerns the underlying mechanisms of the occipital cortex reorganization in the EB subjects: which developmental brain mechanisms do allow blind humans to process non visual stimuli using their “visual” cortex? The study of olfactory perception by the blind contributes in a very original and important way to previous knowledge about the neural processes that are responsible for intermodal plastic changes in the visually deprived occipital cortex. It is generally agreed that two types of mechanisms (and their related neural pathways) may be involved in the functional brain reorganization after blindness and could mediate the functional recruitment of occipital brain areas in non-visual information processing. The model generally favored by the neuroscientists who use neuroimaging techniques predicts that a major cortico-cortical reorganization allows non visual activation of occipital brain areas through the functional recruitment of cortico-cortical connections between auditory cortex, somatosensory cortex or supramodal brain areas and visually deprived occipital brain regions. These cortico-cortical connections would also exist, but would be generally masked in sighted subjects, due to the concurrent stimulation of functional visual connections and these crossmodal sensory connections (Bavelier and Neville, 2002; Pascual-Leone et al., 2005). Another model predicts that a functional brain reorganization due to early blindness involves mainly subcortical structures, the thalamus in particular, leading to functional changes in the subcortical afferents (i.e., the thalamo-cortical connections in particular) that transmit sensory information to cortical brain areas, among which the visually deprived occipital cortex (Bavelier and Neville, 2002; Burton, 2003; Coullon et al., 2015). In the debate opposing the two models, the evidence of a functional recruitment of occipital brain areas during olfactory processing by EB subjects brings at first glance strong support to the cortico-cortical reorganization model since the olfactory system has an exceptional characteristic: the absence of a thalamic intermediary between olfactory epithelium and primary olfactory cortex (Hummel and Welge-Lüssen, 2006). In other words, in contrast to the other sensory modalities, all olfactory information passes directly from the OBs to the primary olfactory brain areas, without relay and modulation in the thalamus, unlike the visual, auditory, and tactile information (Hummel and Welge-Lüssen, 2006; see also Royet and Plailly, 2004). Here, we referenced a number of studies showing that the processing of olfactory information by EB subjects activated their occipital brain areas. Although this might be considered as a strong argument against the thalamic reorganization in blindness, we feel that a more rigorous demonstration of olfactory inputs to occipital cortex is needed to sustain this assertion. It should be noted that, although the secondary olfactory cortex receives its main afferents directly from the primary olfactory cortex, the orbitofrontal cortex and the insula also receive indirect inputs from the piriform cortex and olfactory tubercle that relay in the thalamus before (Royet and Plailly, 2004; Wilson et al., 2006). In addition, if we consider that the main odor stimuli have both olfactory and trigeminal properties, some trigeminal information could be processed in the thalamus before transmission to the somatosensory cortex during an olfactory task (Brand, 2006; Boyle et al., 2007). In our fMRI study (Renier et al., 2013), we used four chemical odorants, among which banana (i.e., iso-amyl-acetate) that is considered as a bimodal olfactory-trigeminal stimulus (Lombion et al., 2009). For these reasons, the evidence of strong occipital cortex recruitment by olfactory processing in EB humans does not allow us to reject formally the hypothesis of cortico-subcortical reorganization as the underlying mechanism responsible for occipital cortex activation in non visual processing by EB subjects. We propose to consider the fMRI studies (Kupers et al., 2011; Renier et al., 2013) as pioneer studies in this context, bringing support to the cortico-cortical reorganization model, whereas additional studies aimed at testing possible differences between olfactory and trigeminal stimulation (i.e., contrasting pure olfactory odorants with trigeminal odorants and taking into account their specific properties) should further assess this point.

As mentioned above, according to the cortico-cortical reorganization model, a potential source of the occipital cortex recruitment in EB subjects is the existence of neural connections between this cortex and the several cortical brain areas related to the remaining senses (Merabet and Pascual-Leone, 2010). Previous studies in animals proved the existence of projections from auditory cortical areas to visual cortical areas (Falchier et al., 2002; Rockland and Ojima, 2003). There are also previous studies in sighted humans that provided evidence of visual brain areas receiving non-visual inputs and being involved in tactile processing (e.g., Sathian et al., 1997; Zangaladze et al., 1999; Amedi et al., 2001; Hagen et al., 2002; James et al., 2002; Merabet et al., 2004; Prather et al., 2004; Kitada et al., 2009, 2014) and auditory processing (Cate et al., 2009). There is also evidence of a cross-modal recruitment of visual brain areas in sighted subjects who experienced transitory visual deprivation (Pascual-Leone and Hamilton, 2001; Merabet et al., 2008). All together these observations indicate that a functional reorganization of existing cortico-cortical connections could drive non-visual information to the visually deprived occipital cortex of EB subjects, contributing to their enhanced abilities in the remaining senses. In the case of olfactory perception, there are previous studies in sighted subjects that showed activation of the lingual gyrus and the cuneus during odor identification (Qureshy et al., 2000; Suzuki et al., 2001), odor edibility and odor hedonicity judgments (Royet et al., 1999, 2001). In addition, odor familiarity judgments activated the mid-FG and the superior occipital gyrus (Plailly et al., 2005). Our own observations are in accordance with these results, since we also observed some activation of visual brain areas in the SC group during odor processing tasks (Renier et al., 2013). According to the main authors, the activation foci in the occipital cortex of sighted individuals would be caused by visual imagery of objects induced by odor perception (Qureshy et al., 2000; Royet et al., 2001) although visual brain areas might also be involved in hedonic judgments and semantic processing of odors (Royet et al., 1999; Plailly et al., 2005). Notwithstanding the limitations we mentioned above, the fMRI studies described in the present review provided convincing data concerning the functional involvement of early deprived visual cortex in olfactory processing. Although we may not exclude a thalamic contribution, we favor the hypothesis according to which visual deprivation would lead to enhanced cortico-cortical connectivity and to unmasking and reinforcement of pre-existing connections between the occipital cortex and olfactory brain areas, allowing cross-modal plastic changes and a reattribution of function to the occipital cortex in EB subjects. The overall idea that emerged from these studies is that, in the absence of visual inputs, non visual sensory modalities including olfaction extend their brain networks into the occipital cortex to improve perceptual and cognitive processing and do so in a specific way, leading to a functional specialization of the occipital cortex between non visual senses. To sustain this claim, additional neuroimaging studies testing multisensory cognitive tasks in the same EB subjects should further investigate to what extent different sensory modalities, among which olfaction, are segregated in their occipital cortex and how non visual inputs promote development of functional modules within the “visual” brain areas.

In conclusion, behavioral adjustments of EB subjects through non visual sensory modalities also apply to olfaction. Superior olfactory abilities are present at all levels of olfactory stimulus processing (i.e., sensory, basic perceptual and cognitive processing). However, these superior olfactory abilities are especially evident when the tasks are complex, including such cognitive components as semantic memory. Although additional studies are clearly needed, the referenced studies indicate that subjects with early-onset blindness could make a larger use of odorous stimuli than sighted individuals to compensate for the lack of vision. Passive olfactory stimulation produces a similar intermodal activation of occipital brain areas in EB and SC subjects, whereas there is a significantly higher recruitment of the occipital cortex in blind subjects compared to SCs during active odor detection and higher-level cognitive processing of odors. Additional neuroimaging investigations comparing the effect of pure olfactory and trigeminal odorants, as well as contrasting the orthonasal and retronasal ways, are clearly needed to elucidate how this cross-modal activation of the occipital cortex contributes to olfactory processing in EB subjects, since these investigations will help to elucidate the contribution of cortico-cortical connections in the functional reorganization of occipital cortex in EB subjects, which remains a central question in the field of brain plasticity and blindness rehabilitation.

## Author Contributions

AGDV designed referenced studies, acquired and analyzed the data and wrote the article. RA wrote the article with AGDV. LAR, IC and PR participated in data acquisition, analysis and writing process.

## Conflict of Interest Statement

The authors declare that the research was conducted in the absence of any commercial or financial relationships that could be construed as a potential conflict of interest.
